# Supplementary material for: Coinfection of Two Rickettsia Species in a Single Tick Species Provides New Insight into Rickettsia-Rickettsia and Rickettsia-Vector Interactions
Source: Microbiol Spectr. 2022 Sep 29;10(5):e02323-22. doi: 10.1128/spectrum.02323-22 (PMC9603609; doi:10.1128/spectrum.02323-22)
Supplement: Supplemental file 1 — Supplemental material. Download spectrum.02323-22-s0001.pdf, PDF file, 1.2 MB [file spectrum.02323-22-s0001.pdf]

SUPPLEMENTAL MATERIAL

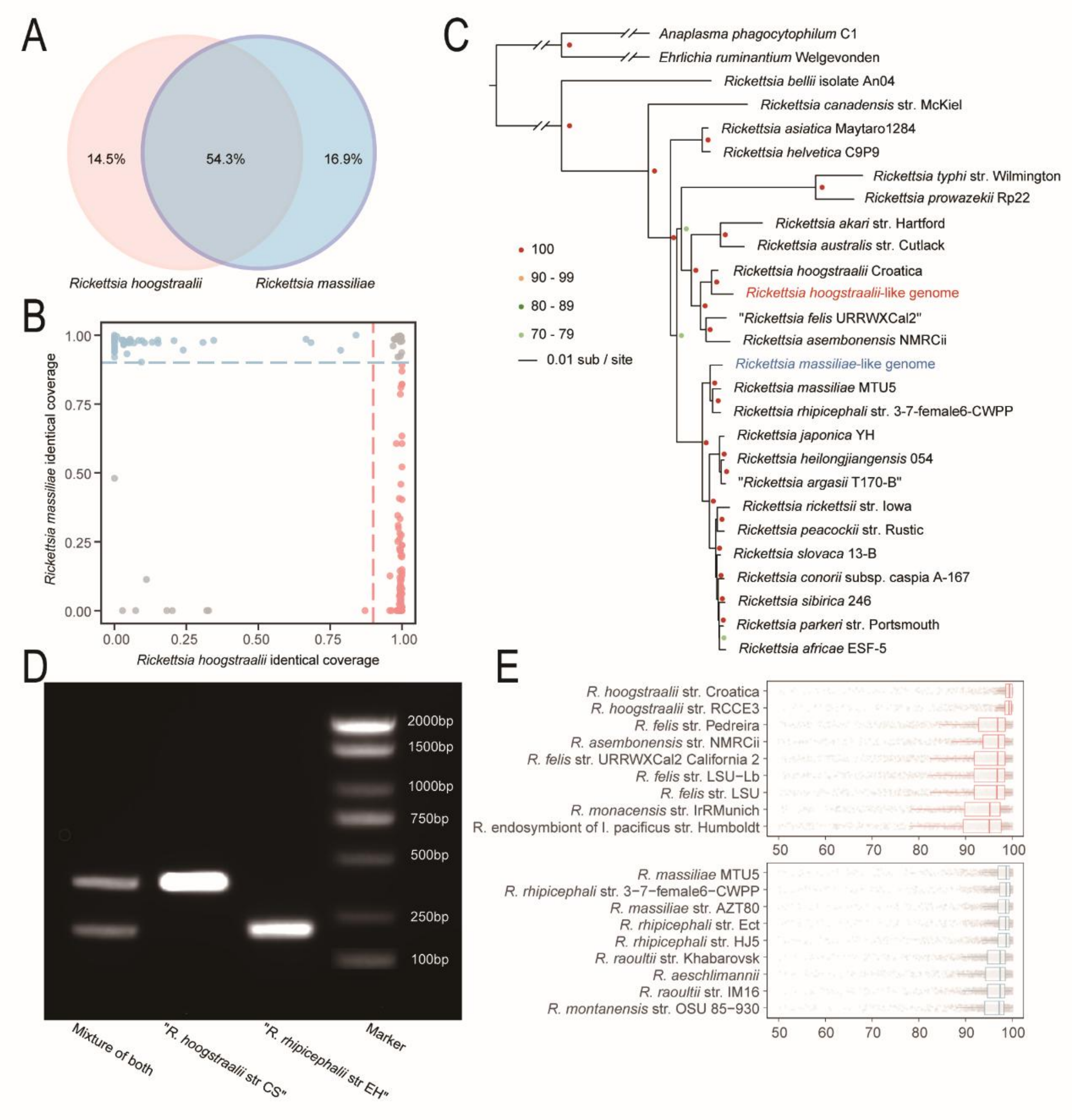

FIG S1 Confirmation of the methods for determining co-occurrence of two novel *Rickettsia*, related to Figure 1.

(A) Venn plot of proportions of reads that can be mapped to *Rickettsia hoogstraalii* Croatica (pink) and *R. massiliae* MTU5 (blue).

(B) Identical coverage of each of the contigs assembled from the *R. massiliae*-like isolate to *R. hoogstraalii* Croatica and *R. massiliae* MTU5 genomes. Identical

coverage was calculated according to identical length divided by contig length. Blue points: contig probably came from a *Rickettsia* species closely related to *R. massiliae*; pink points: contig probably came from a *Rickettsia* species closely related to *R. hoogstraalii*; dark gray points: contig probably came from overlapping sections of the two *Rickettsia* genomes; light gray points: contig probably came from novel sections of the two *Rickettsia* genomes.

(C) Maximum likelihood phylogenomic tree of *Rickettsia hoogstraalii*-like genome and *Rickettsia massiliae*-like genome with 25 other publicly-available Rickettsiales species. Tree was inferred by Raxml based on 317 single copy orthologs identified by orthofinder. 1,000 alternative runs were used to calculate support values. *Anaplasma phagocytophilum* and *Ehrlichia ruminantium* were two outgroup species to help root the tree.

(D) Specific primers were used to amplify the *ompA* gene in a mixture of the two novel *Rickettsia* spp. *"R. hoogstraalii* str CS*"* and *"R. rhipicephalii* str EH" (mix), *"R. hoogstraalii* str CS*"* alone and "*R. rhipicephalii* str EH*"* alone.

(E) All-to-all protein Blastp hits of "*"R. hoogstraalii* str CS*"* (top) and "*R. rhipicephalii* str EH*"* (bottom) to the top nine most closely related rickettsiae species.

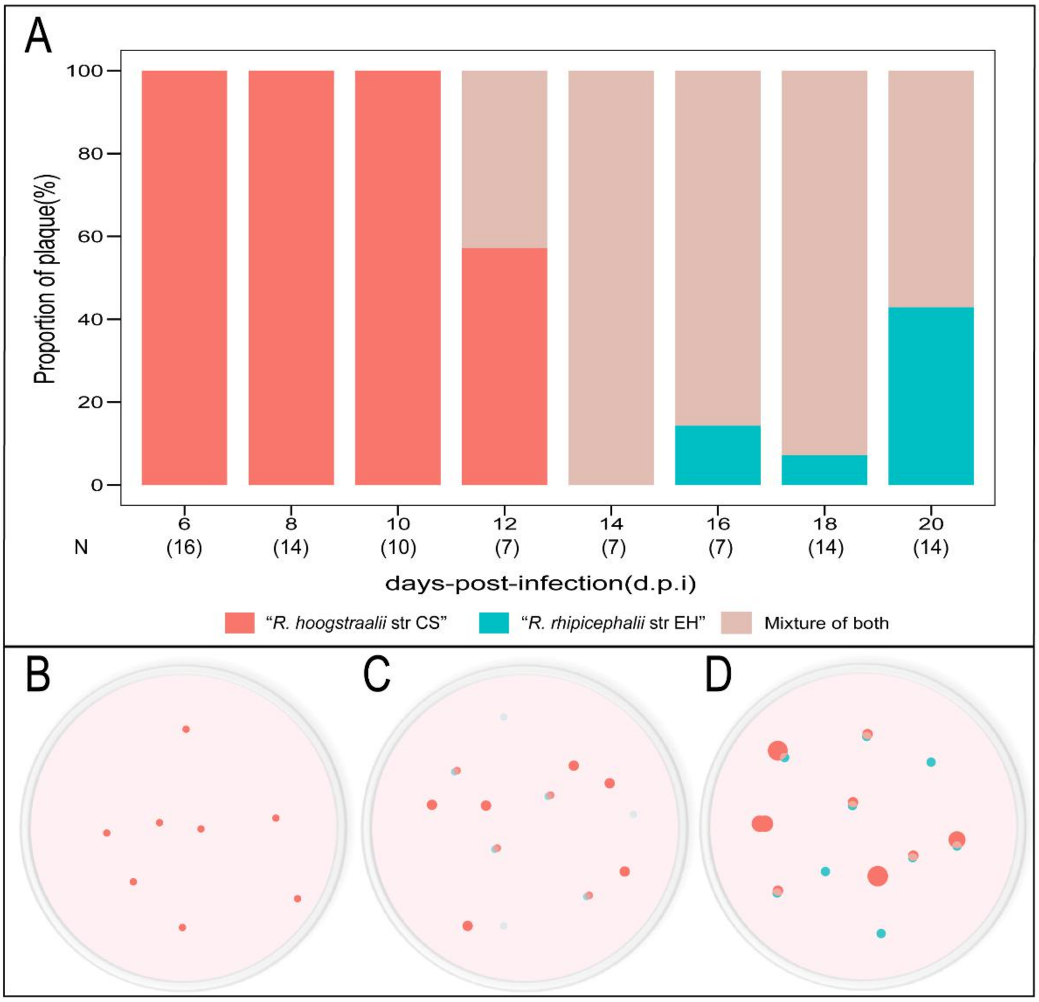

FIG S2 (A) The proportion of plaques formed in Vero 81 cells by the two distinct rickettsiae in mixed populations at different times, related to Figure 1. n = 89. The schematic drawing of plaque formation: (B) between 6-10 days post infection (d.p.i.) almost all plaques were *"Rickettsia hoogstraalii* str CS", (C) and (D) from 12 to 20 d.p.i., the plaques were larger and the percentage formed by "*Rickettsia rhipicephalii* str EH*"* increased. The coloring of the plaques in the schematic plates is based on PCR results of sampling plaques.

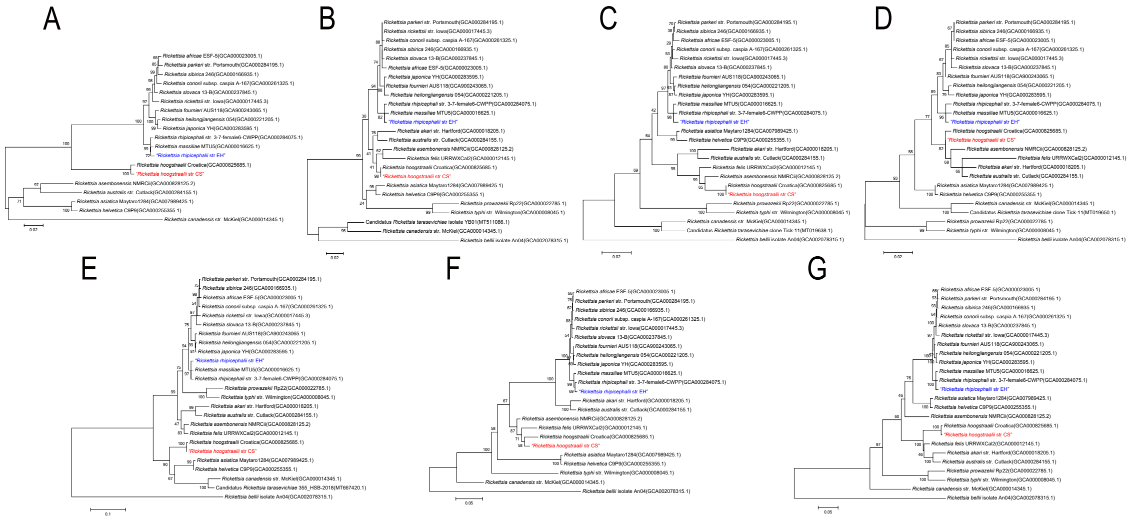

FIG S3 Phylogenetic analysis of concatenated nucleotide sequence of "*R. hoogstraalii* str CS" and "*R. rhipicephalii* str EH". The partial nucleotide sequences of genes ompA(A), 17kDa(B), gltA(C), groel(D), ompB(E), sca1(F) and sca4(G) were concatenated and compared via the maximum-likelihood method by using the best substitution model (i.e., Kimura 2-parameter model) and MEGA version 6.0 (http://www.megasoftware.net). A bootstrap analysis of 1,000 replicates was applied to assess the reliability of the reconstructed phylogenies, and bootstrap values are indicated at branch nodes. Scale bar indicates the number of substitutions per nucleotide position.

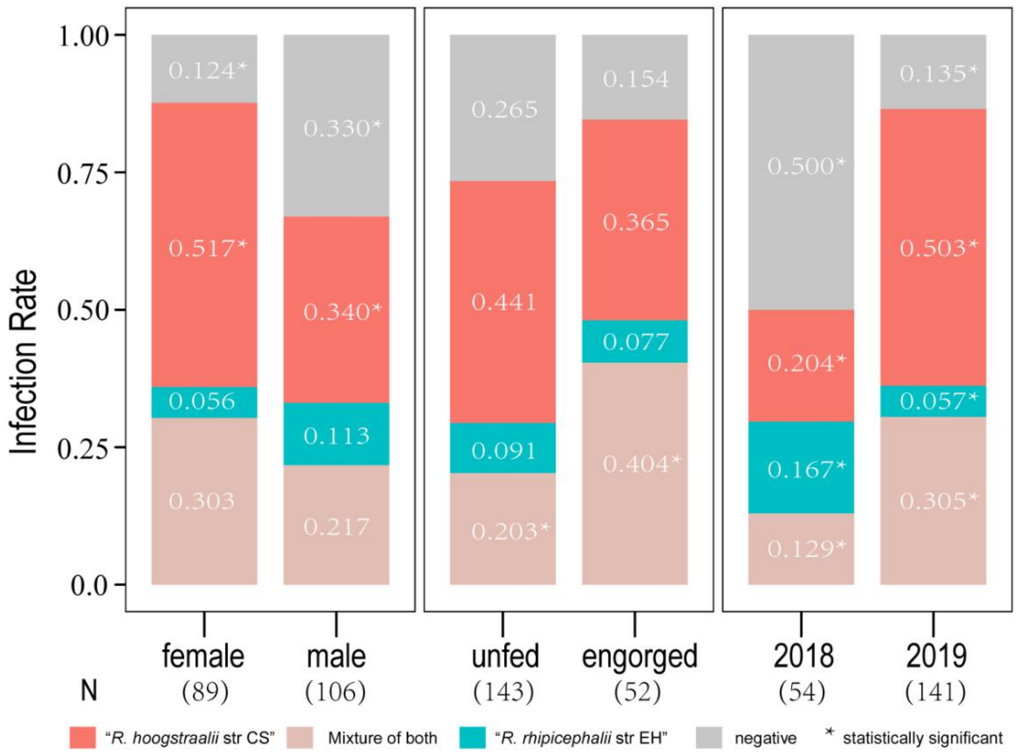

FIG S4 Natural Infection Rates of "*Rickettsia hoogstraalii* str CS*"* and "*Rickettsia rhipicephalii* str EH*"* in Field-collected Ticks: The infection rates of *Hauemaphysalis montgomeryi* (n = 195) based on male/female, unfed/engorged and time of collection. N represents the number of samples. A total of 195 *H. montgomeryi* adult ticks were collected from Dali City and were tested by the specific PCR. The engorged ticks were all detached from the goats in the field.

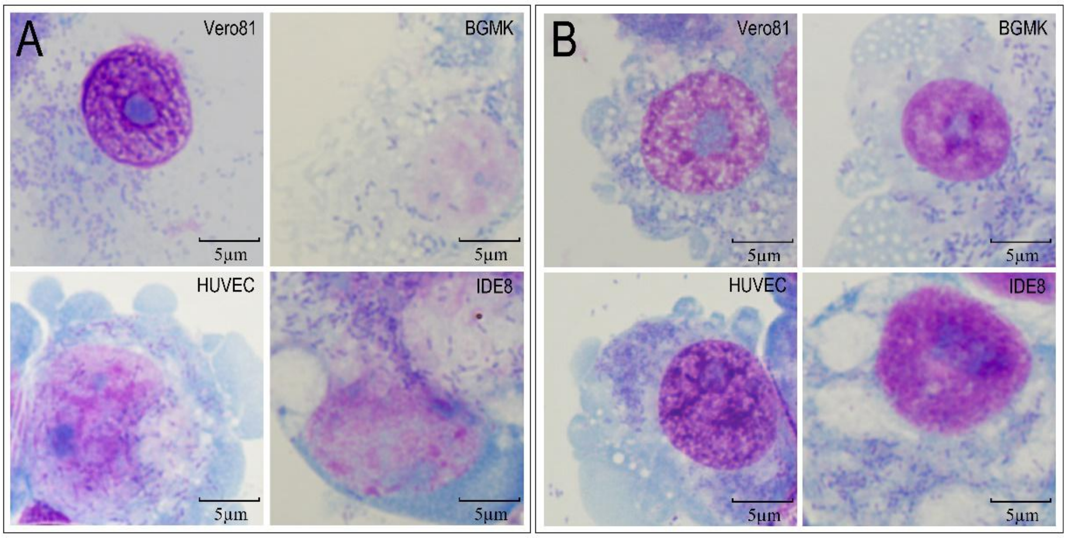

FIG S5 Giemsa staining of two novel *Rickettsia* in the cell lines Vero 81, HUVEC, BGMK and IDE8: (A) *"Rickettsia hoogstraalii* str CS*"*. (B) "*Rickettsia rhipicephalii* str EH"*,* related to Figure 2

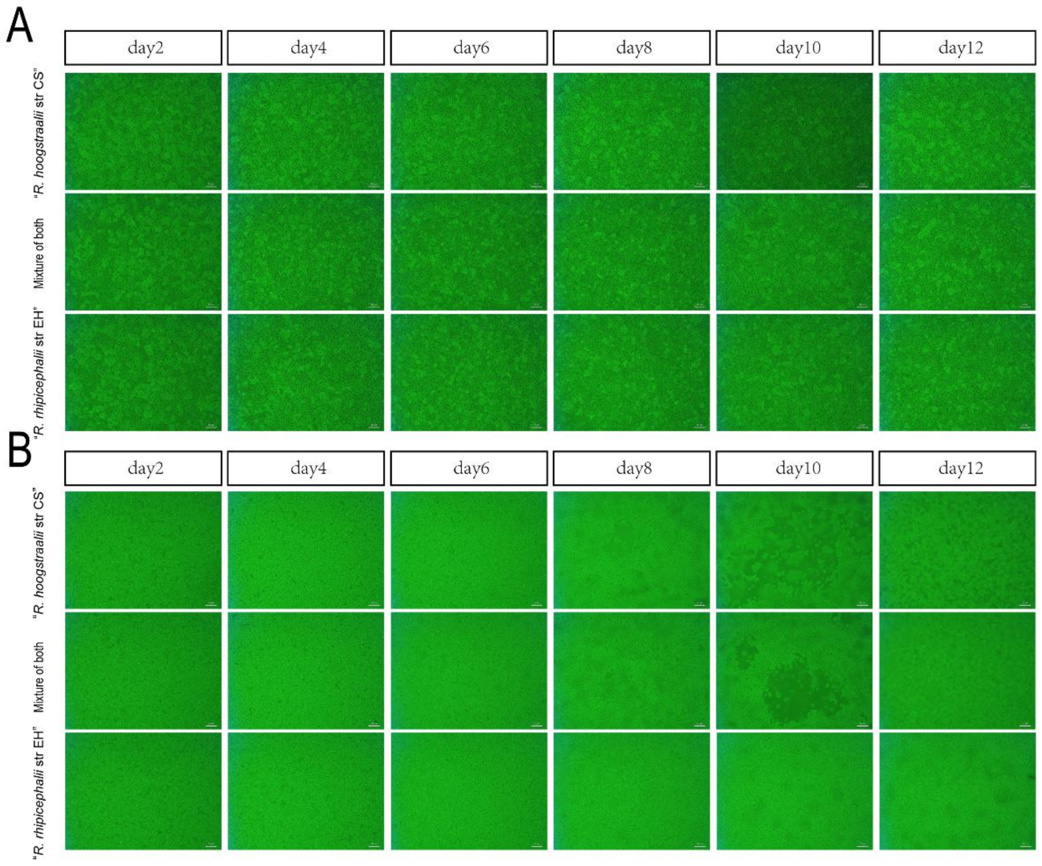

FIG S6 Cytopathic effect (CPE) in HUVEC (A) and IDE8 (B) cells induced by *"Rickettsia hoogstraalii* str CS*"* "*Rickettsia rhipicephalii* str EH*"* or a mixture of both, related to Figure 2.

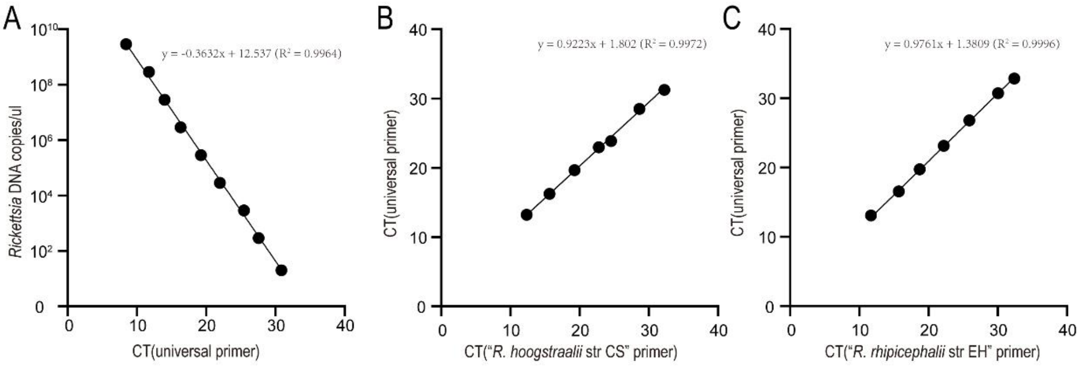

FIG S7 Primers validated to reliably quantify the relative amounts of two rickettsiae in a mixed sample in the competition assay, related to Figure 3. Universal primers could amply both *"Rickettsia hoogstraalii* str CS*"* and "*Rickettsia rhipicephalii* str EH*"*; *"R. hoogstraalii* str CS" primer was specific for *"R. hoogstraalii* str CS*"* while *"R. rhipicephalii* str EH*"* primer was specific for *"R. rhipicephalii* str EH*"* (Table S1)

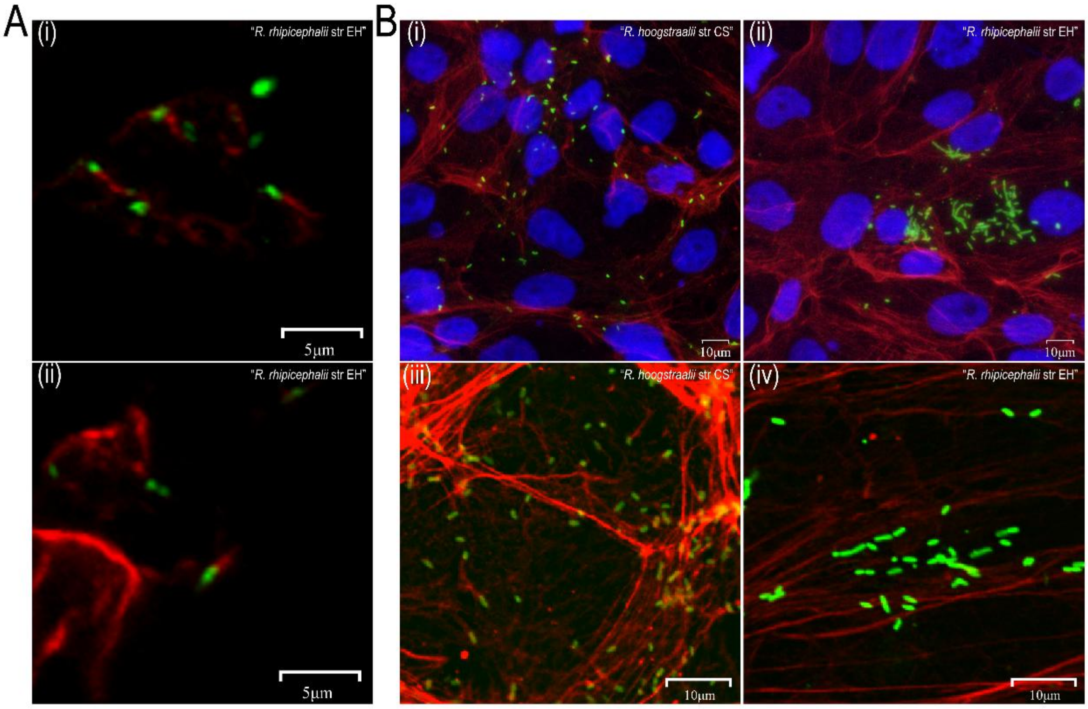

FIG S8 Actin tail phenotype of *"Rickettsia hoogstraalii* str CS*"* and *"Rickettsia rhipicephalii* str EH*" in vitro*, related to Figure 4.

(A) Actin tail phenotype of *"R. rhipicephalii* str EH*"* in HUVEC cells only seen at occasional confocal scanning for 96 hr post infection.

(B) Actin comet tail formation by *"Rickettsia hoogstraalii* str CS*"* and *"Rickettsia rhipicephalii* str EH*"* in Vero 81 cells:(i) *"R. hoogstraalii* str CS*"* were scattered while (ii) *"R. rhipicephalii* str EH*"* were concentrated in the infected cells (iii) *"R. hoogstraalii* str CS*"* actin tails were long and unbranched at 4 days post-infection (d.p.i.) (iv) "*R. rhipicephalii* str EH*"* actin tails were not obvious either at 2 or 4 d.p.i..

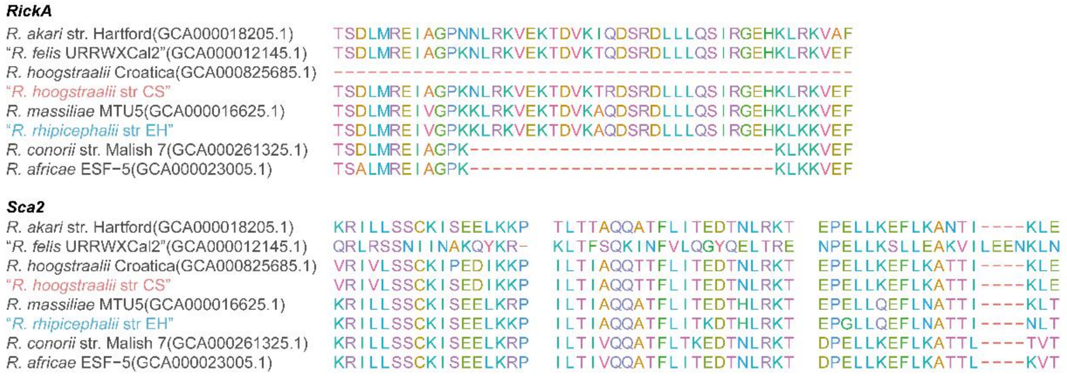

FIG S9 Alignment of key protein motifs associated with rickettsial actin-based motility for *"Rickettsia hoogstraalii* str CS*"* and *"Rickettsia rhipicephalii* str EH". related to Figure 4.

Top, RickA WASP-homology 2; and bottom, Sca2 putative WH2 motifs.

TABLE S1 List of universal and specific primers designed for *"Rickettsia hoogstraalii* str CS*"* and "*Rickettsia rhipicephalii* str EH".

Rickettsia	Primers	Seq (5' -3')	Length
"*R. hoogstraalii* str CS"	YN1-*ompA*122	5' CATCGTCATCACCGTCTA 3'	391bp
	YN1-*ompA*512	5' GCTAATGGTAATCCTGCT 3'	
"R. rhipicephalii str EH"	YN2-*ompA*115	5' GTTATTATACCTCCTCCATC 3'	209bp
	YN2-*ompA*323	5' TTGCCTGTTACTATTACTGC 3'	
Universal primer	*ompA*20	5' TAACTTAACAGGCAGCATA 3'	100bp
	*ompA*119	5' ATTAGCCGCAGTCCCTAC 3'	

⋆ Universal primer for both "*R. hoogstraalii* str CS" and "*R. rhipicephalii* str EH"

TABLE S2 Transstadial Transmission of Co-infection with "*Rickettsia hoogstraalii* str CS*"* and "*Rickettsia rhipicephalii* str EH*"* between nymphal and adult stages of *Haemaphysalis montgomeryi* ticks.

parameters	*Haemphysalis montgomeryi* colonies		
	"R. hoogstraalii str CS"	*"R. rhipicephalii* str EH*"*	Co-infection
Engorged nymph weight (n)	3.05mg (13)	3.35mg (11)	3.25mg (10)
Adult tick weight (n)	1.95mg (13)	1.94mg (10)	2.02mg (10)
Full engorgement rate	92.9% (13/14)	84.6% (11/13)	90.9% (10/11)
Molting rate	100% (13/13)	90.9% (10/11)	100% (10/10)
